# Optogenetic Long-Term Manipulation of Behavior and Animal Development

**DOI:** 10.1371/journal.pone.0018766

**Published:** 2011-04-20

**Authors:** Christian Schultheis, Jana Fiona Liewald, Ernst Bamberg, Georg Nagel, Alexander Gottschalk

**Affiliations:** 1 Institute of Biochemistry, Goethe-University, Frankfurt, Germany; 2 Frankfurt Institute for Molecular Life Sciences (FMLS), Goethe-University Frankfurt, Frankfurt, Germany; 3 Department of Biophysical Chemistry, Max-Planck-Institute of Biophysics, Frankfurt, Germany; 4 Botanik I, University of Würzburg, Würzburg, Germany; Yale School of Medicine, United States of America

## Abstract

Channelrhodopsin-2 (ChR2) is widely used for rapid photodepolarization of neurons, yet, as it requires high-intensity blue light for activation, it is not suited for long-term *in vivo* applications, e.g. for manipulations of behavior, or photoactivation of neurons during development. We used “slow” ChR2 variants with mutations in the C128 residue, that exhibit delayed off-kinetics and increased light sensitivity in *Caenorhabditis elegans*. Following a 1 s light pulse, we could photodepolarize neurons and muscles for minutes (and with repeated brief stimulation, up to days) with low-intensity light. Photoactivation of ChR2(C128S) in command interneurons elicited long-lasting alterations in locomotion. Finally, we could optically induce profound changes in animal development: Long-term photoactivation of ASJ neurons, which regulate larval growth, bypassed the constitutive entry into the “dauer” larval state in *daf-11* mutants. These lack a guanylyl cyclase, which possibly renders ASJ neurons hyperpolarized. Furthermore, photostimulated ASJ neurons could acutely trigger dauer-exit. Thus, slow ChR2s can be employed to long-term photoactivate behavior and to trigger alternative animal development.

## Introduction

ChR2 is a light-driven cation channel that enables fast photodepolarization of excitable cells in culture and in live animals ranging from *Caenorhabditis elegans* to primates [1]–[6]. However, for long-term photodepolarization, e.g. to influence learning or neuron-controlled alternative developmental pathways, ChR2 is not suited: As it requires continuous illumination with blue light of high intensity (≥1 mW/mm^2^) to keep the channel open a) phototoxicity may arise and b) intrinsic phototactic reactions of animals can occur that interfere with the studied behavior. These limitations may be overcome by the recently described ChR2(C128X) mutants [7]–[9]. Compared to wild type ChR2 (τ_off_ = 11.9 ms), mutations of C128 to T, A, or S significantly delay the closing of the channel in the dark (τ_off_ = 2 s, 56 s, and 106 s, respectively; [7]). As the open photointermediate P520 accumulates, light of reduced intensity suffices for efficient channel-opening. Once in the open state, C128X mutants can be photoinactivated using green-yellow light, thus they are also termed “step function opsins”.

*C. elegans* is a genetic model for studies of neurobiology and development, among other areas of biology. Its nervous system is mapped down to the individual synapse [10], and its neurons form simple functional units, similar to elementary network units found in higher animals [11]. *C. elegans* exhibits stereotypic behaviors, e.g. escape reflexes in response to particular sensory inputs, and, depending on external conditions, alternative developmental pathways. In a favorable environment, the nematode develops through four larval stages into adult animals [12], while under harsh conditions, reproductive development is bypassed and animals enter a long-lived “dauer”-state after larval stage L2 [13]–[15]. Dauer larvae exhibit specialized morphology and metabolism, allowing them to survive harsh conditions for several months [13], [16]–[18]. Importantly, harsh or beneficial conditions are detected by sensory neurons that prevent or instruct entry into, or exit from, the dauer-state [19], [20].

We characterized slow ChR2 variants for prolonged photoactivation of excitable cells in *C. elegans*. ChR2(C128X) could photodepolarize body wall muscle (BWM) cells, cholinergic and GABAergic motorneurons for several minutes following a 1 s light pulse. As in other systems, the open state could be terminated by yellow light. Continuous activation of the locomotion command interneurons evoked long-lasting behavioral alterations. Lastly, we could alter the genetically predisposed development of *C. elegans* by long-term photodepolarization of ASJ sensory neurons, to either prevent the constitutive dauer-entry in *daf-11* mutant animals, or to achieve an exit from the dauer-state.

## Results

### Slow ChR2 mutants enable long-term activation of muscles with low light intensity

**Figure 1:**
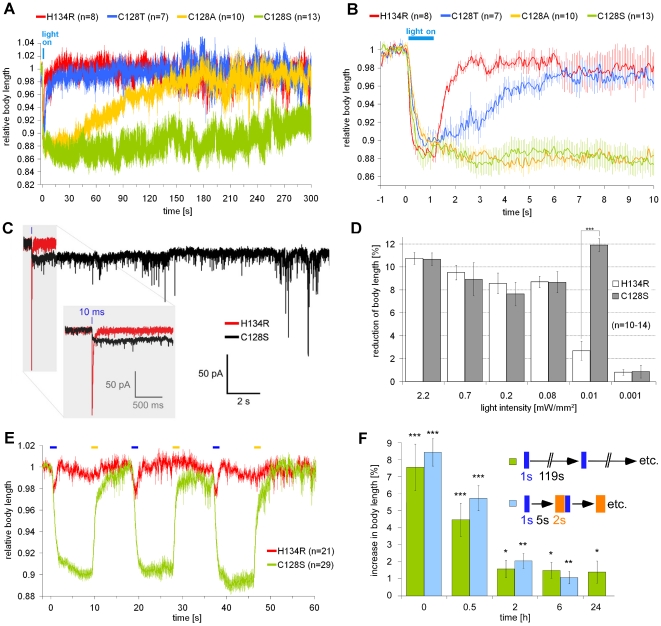
ChR2(C128X) variants induce prolonged depolarization in muscles at reduced light intensities. (**a**) ChR2(H134R), ChR2(C128A, T and S) were expressed in BWMs. Body length of animals during and after a 1 s blue light stimulus (0.69 mW/mm^2^; 450–490 nm; given at t = 0 s) was calculated relative to the initial length. (**b**) Enlarged (seconds -1 to 10) from (a). (**c**) Representative traces of photocurrents measured in whole-cell voltage clamp of BWMs expressing ChR2(C128S) or ChR2(H134R), evoked by a 10 ms photostimulus (470 nm; 8 mW/mm^2^); inset shows close-up. (**d**) Comparison of the light-dependence of ChR2(H134R) and ChR2(C128T) in BWMs. Reduction of body length of worms in response to 1 s blue light stimuli (450–490 nm) of various light intensities in the range of 0.001–2.2 mW/mm^2^, presented at t = 0 s; reduction of body length was measured for ChR2(H134R) directly after light off (t = 1 s), for ChR2(C128S), due to the slower onset, 2 s after light off (t = 3 s). (**e**) Relative body length of worms while alternating 1 s blue (0.01 mW/mm^2^; 450–490 nm) or 1 s yellow (4.4 mW/mm^2^; 565–595 nm) light pulses were presented, indicated by blue and yellow bars. (**f**) Long-term activity of ChR2(C128S). 1 s blue light pulses (0.05 mW/mm^2^; 470 nm) were presented either every 120 s or every 8 s (in the latter case, followed by a 5 s dark period and a 2 s yellow light pulse (0.04 mW/mm^2^; 590 nm)). At the indicated times, animals (n = 9–15) were presented a blue test pulse (2.1 mW/mm^2^; 450–490 nm), followed by a yellow pulse (6.1 mW/mm^2^; 565–595 nm) for inactivation and the resulting relaxation was measured. n = number of animals; error bars are s.e.m.; ***: p<0.001, **: p<0.005, *: p<0.01.

First, we expressed three slow ChR2 variants (C128T, A, and S), in BWMs (**Fig. S1a**), as well as ChR2(H134R), which has slightly delayed kinetics and thus larger steady-state conductance than wild type ChR2 [4]. Concomitant depolarization of all BWMs induces a uniform contraction, causing a decrease in body length; thus, body length is a measure for extent and persistence of BWM depolarization [4]. The proteins localized mostly to the plasma membrane, with variable amounts of intracellular, sometimes aggregated protein. We analyzed expression levels based on fluorescence in individual muscle cells, which showed strong differences: ChR2(C128T and S) both expressed better than ChR2(H134R), while ChR2(C128A) expression was low, showing mosaicisms (data not shown). However, as the proteins aggregated to a variable extent and we could not specifically determine cell surface expression levels, these findings did not allow us to predict which protein may be best suited for long-term applications in *C. elegans*. Thus, to assess this based on function, we monitored the capability of the ChR2 variants to depolarize BWMs. Animals (grown in presence of all-*trans* retinal - ATR) were illuminated for 1 s with blue light (450–490 nm; 0.69 mW/mm^2^), and the body length was deduced from videos [5] (**Fig. 1a, b** **, and Video S1**). ChR2(H134R) induced a ∼12% contraction, and animals returned to initial length ∼1 s after light-off. ChR2(C128T, A, and S) induced comparable contraction amplitudes, however, relaxation of the body wall was largely delayed, occurring after 5 s, 3 min, and >5 min, for ChR2 C128T, C128A, and C128S, respectively. Measuring ChR2(C128S)-mediated inward photocurrents confirmed the largely delayed channel-closing (**Fig. 1c**, and see below).

We determined the lowest light intensity (0.001–2.2 mW/mm^2^) sufficient to achieve saturating ChR2-dependent effects. Contractions >8% were evoked by ChR2(H134R) in BWMs when at least 0.08 mW/mm^2^ light were applied. In contrast, photoactivation of ChR2(C128S) with as low as 0.01 mW/mm^2^ still evoked full contractions (**Fig. 1d**), that were also prolonged for several minutes (**Fig. S1b, c**), thus establishing ChR2(C128S) as a powerful tool for prolonged depolarization of excitable cells under minimal light-invasive conditions. As daylight already caused marked contraction and uncoordinated locomotion, ChR2(C128S) animals should be kept in the dark and handled under low intensity red light to prevent unwanted photoactivation.

### ChR2(C128S) can be repeatedly “switched” on and off with blue an yellow light

As in other systems, we could photo-switch slow ChR2 variants from the open state to the closed dark-state, by using yellow light [7]. We applied alternating blue (450–490 nm; 1 s; 0.01 mW/mm^2^) and yellow (565–595 nm; 1 s; 4.4 or 2.5 mW/mm^2^) pulses, each followed by an 8 s dark period, and monitored the body length of animals expressing either ChR2(H134R) or ChR2(C128S) in BWMs (**Fig. 1e** **and Video S1**). In H134R animals, low intensity blue light induced ∼2% contraction during illumination, while yellow light had no effect. In contrast, in C128S animals, blue light induced a continuous contraction of ∼10% that was completely abolished by the yellow light pulse (**Fig. 1e**), thus allowing full temporal control over ChR2(C128S) induced depolarization.

### Long-term stimulation of ChR2(C128S) leads to a partial reduction of function

Potential applications of ChR2(C128S) could be to keep neurons depolarized for hours to days to affect processes like learning or even developmental pathways. However, Schoenenberger et al. (2009) found a progressive inactivation of ChR2(C128A), when in the open state, and in response to repeated stimuli. A fraction of molecules appeared to transition into an ill-defined, non-activatable “lost state”, from which they recovered very slowly. We thus assayed for how long ChR2(C128S) may be continuously activated. ChR2(C128S) in muscles was photoactivated for up to 1 day using two different protocols: a) 1 s blue light every 2 min; or b) 1 s blue, 5 s dark, then 2 s yellow, etc., the latter one to actively prevent loss of ChR2 to inactive states. At 0, 30, 120, 360 minutes and 1 day, animals were given a blue test pulse for full activation, followed by a yellow pulse for inactivation, and the relaxation was measured (**Fig. 1f**). After 30 min, effects were reduced from initially 7.6±1.4% to 4.5±1.0%, after 120 min, and still after 1 day, they were down to 1.4±0.6%. Thus, long-term depolarization via ChR2(C128S) may cause only ∼18% of maximal effects and should be considered when using ChR2(C128S) in the range of several hours or days. Nevertheless, depending on the cell type, this remaining functionality for even 24 h may be sufficient for long-term activation of the particular cell and its potential downstream targets.

### Slow ChR2 mutants allow activating motorneurons in *C. elegans*

**Figure 2:**
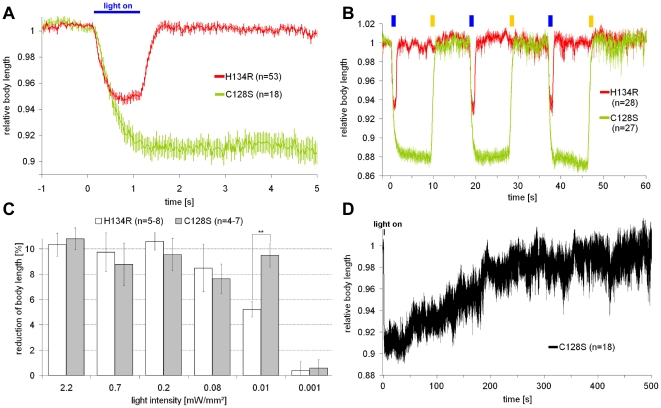
ChR2(C128S) induces prolonged depolarization in cholinergic motorneurons at reduced light intensities. ChR2(H134R) and ChR2(C128S) were expressed in cholinergic motorneurons. To avoid coiling induced by prolonged depolarization of cholinergic neurons [5], we analyzed effects of ChR2 activation in *unc-49(e407)* mutants, lacking GABA_A_Rs. (**a**) Relative body length of worms while a 1 s blue light stimulus (0.01 mW/mm^2^; 450–490 nm) was given at t = 0 s. (**b**) Relative body length of worms while alternating 1 s blue (0.01 mW/mm^2^; 450–490 nm) or 1 s yellow (2.5 mW/mm^2^; 565–595 nm) light pulses were presented, as indicated. (**c**) Comparison of the light-dependence of ChR2(H134R) and ChR2(C128S). Reduction of body length of worms while a 1 s blue light stimulus (450–490 nm) of various light intensities in the range of 0.001–2.2 mW/mm^2^ was given at t = 0; reduction of body length was measured for ChR2(H134R) directly after light off (t = 1 s), and for ChR2(C128S) when full amplitudes were reached, 2 s after light off (t = 3 s). (**d**) Relative body length of worms expressing ChR2(C128S) while a low-intensity 1 s blue light stimulus (0.01 mW/mm^2^; 450–490 nm) was given at t = 0 s. n = number of animals; error bars are s.e.m.; **: p<0.005.

We next tested the applicability of slow ChR2 mutants in neurons. We expressed ChR2(C128S) in cholinergic motorneurons, that cause muscle contraction when photostimulated [5]. As photostimulation of cholinergic neurons causes a coiling phenotype, due to concomitant GABA signaling, we analyzed the effects of ChR2 activation in *unc-49(e407)* mutants that lack the muscular GABA_A_R. A 1 s, low-intensity (0.01 mW/mm^2^) light pulse caused prolonged contractions of ∼10%, which were long-lasting (several minutes; **Fig. 2a, d** **, and Video S2**), and could not be achieved using ChR2(H134R). Also for cholinergic neurons, we could photo-switch ChR2(C128S) from the open state to the closed dark-state, using yellow light, and this could be repeated up to 10 times, with no obvious loss of activity (**Figs. 2b** **and S2**). As for muscles, the lowest light intensity sufficient to achieve saturating ChR2-dependent effects in cholinergic neurons was 0.01 mW/mm^2^ (**Fig. 2c**). At this light intensity, ChR2(H134R) appeared to evoke contractions more efficiently in cholinergic neurons than in BWMs (compare **Figs. 1d** **and** **2c**). This may be due to cell-type specific differences in the environment of the channel, affecting its properties, or because contractions evoked by ChR2 in cholinergic motorneurons are effected by ACh release and postsynaptic nAChRs, which may be more efficient, than directly by photocurrents within BWMs. Similar experiments with ChR2(C128S) and ChR2(H134R) in GABAergic motorneurons (evoking body relaxation; [5]) showed qualitatively comparable results (**Fig. S3**), emphasizing the utility of ChR2(C128S) in several neuron types. Thus, ChR2(C128S) can be used to mimic prolonged synaptic transmission at the neuromuscular junction.

### Long-term alteration of behavior in locomotion command interneurons

**Figure 3:**
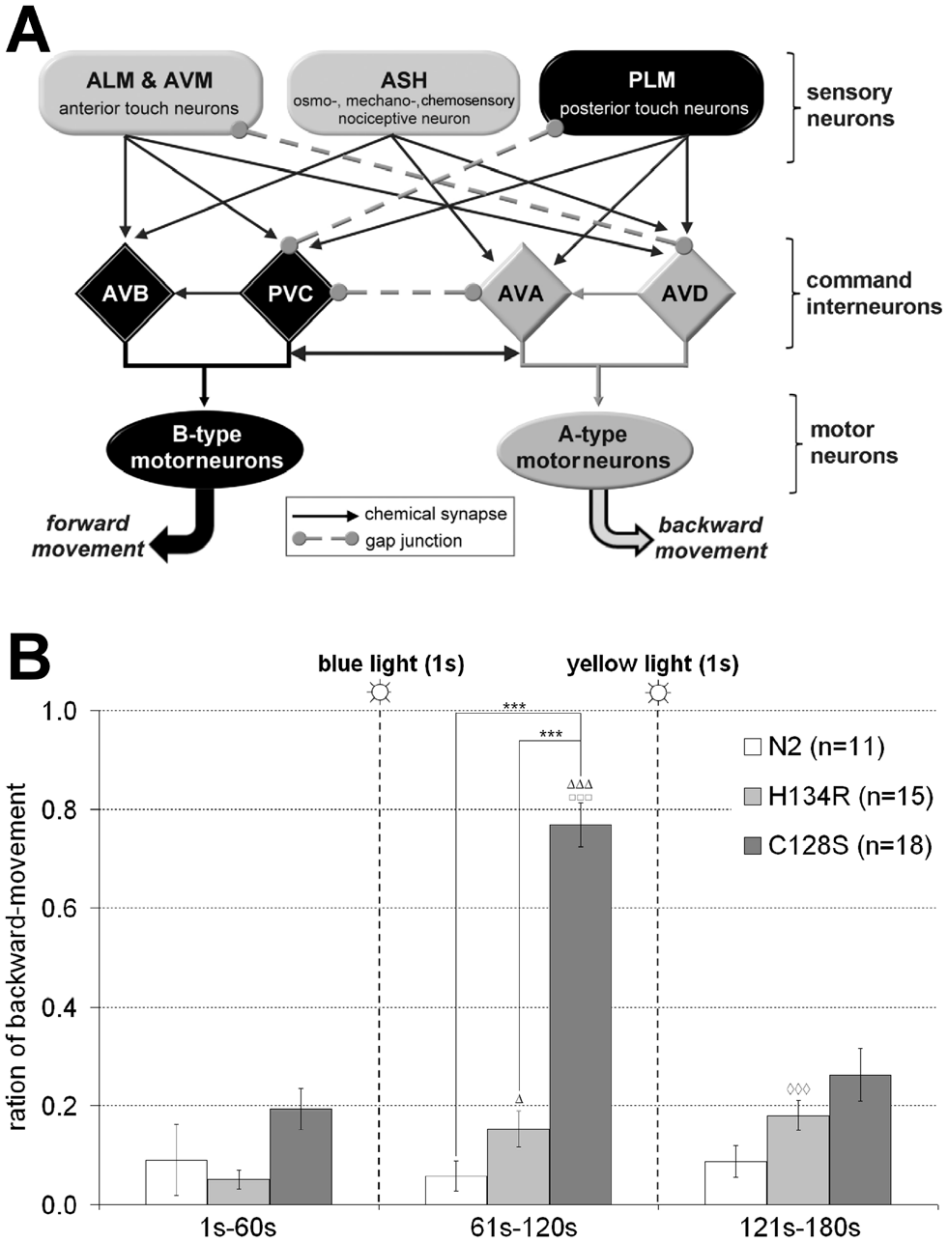
Photoactivated ChR2(C128S) induces long-term behavioral effects in command interneurons. (**a**) Schematic of the neuronal wiring diagram connecting selected sensory neurons, command interneurons and motorneurons that evoke, mediate or are required for forward (indicated in black) or backward locomotion (indicated in grey), respectively. Synaptic strength is omitted, for clarity. (**b**) ChR2(H134R) and ChR2(C128S) were expressed in command interneurons. Animals were placed on NGM plates without food. The ratio of backward locomotion was measured for 3 minutes (binned in 1 min intervals) and compared to wild type animals (N2). After 60 s, a 1 s blue light stimulus (2.1 mW/mm^2^; 450–490 nm) and after 120 s, a 1 s yellow light stimulus (6.1 mW/mm^2^; 565–595 nm) were given. n = number of animals; error bars are s.e.m.; *: significance between different strains; other symbols: significance within a strain between minute 1 and 2 (Δ), minute 2 and 3 (□), or minute 1 and 3 (◊); ***/ΔΔΔ/□□□/◊◊◊: p<0.001, Δ: p<0.01.

We next analyzed whether behavior can be altered in the long-term by depolarizing command interneurons, which regulate certain aspects of locomotion (**Fig. 3a**), particularly the direction and likely also the speed of movement: AVB and PVC neurons trigger forward, whereas AVA and AVD mediate backward locomotion. Each cell type mutually inhibits the opposite type, thus they form a bi-stable switch that fluctuates between backward and forward states. Sensory neuron input alters this balance by depolarizing one command neuron type; in undisturbed animals, forward command neurons dominate, and worms crawl mostly forward, interrupted by brief backward episodes (∼2–4 times min^−1^; [21]). Concomitant activation of all command neurons thus perturbs normal activity and affects locomotion.

We used the *glr-1* promoter to express ChR2(C128S) and ChR2(H134R) in all command neurons and some additional neurons [22] (**Fig. S4**). The ratio of backward movement was assayed for three consecutive 1 min periods, and calculated for the whole period. A 1 s blue light pulse (2.1 mW/mm^2^) was applied after the first minute to activate ChR2 variants. After the second minute, yellow light (1 s; 6.1 mW/mm^2^) was presented for inactivation. Non-transgenic animals (wild type) did not exhibit any significant change in the proportion of backward movement (**Fig. 3b**). For ∼40% of ChR2(H134R) expressing animals, a reversal was observed right after the blue light photoactivation. However, as these effects were very brief, i.e. restricted to the time of illumination, they did not become evident in the analyzed one-minute proportion of backward locomotion. In contrast, ∼71% of ChR2(C128S) animals reversed upon photoactivation and thereafter crawled backwards for extended periods, often even until the inactivating yellow pulse. The proportion of backward movement increased from 19.4±4.2% to 76.8±4.4% during the second minute, which was completely reversed after the inactivating yellow pulse (26.3±5.3%; **Fig. 3b** **and Video S3**). Thus, command neurons can be optically manipulated in the long-term, to evoke downstream effects across several synapses, emphasizing the utility of ChR2(C128S) in prolonged manipulation of neuronal networks and, as a result, behavior.

### Long-term photo-activation of ASJ neurons during animal development

**Figure 4:**
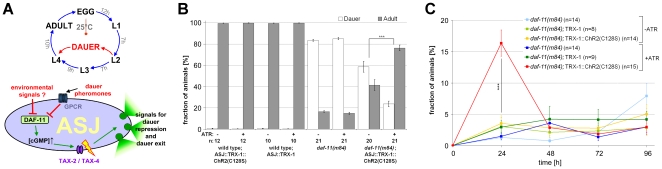
Altering animal development by long-term photostimulation of ASJ neurons. (**a**) Larval development of *C. elegans*, including the alternative dauer-state (upper panel, simplified; [15], [19], [20], [24]–[26], [33]). Sensory neurons, like ASJ, mediate entry into, exit from, or repression of the dauer-state, in response to environmental signals. The molecular mechanisms in ASJ (lower panel, modified; [26]) are depicted. When depolarized, ASJ releases signals causing dauer-repression and dauer-exit. ASJ is depolarized via cGMP-gated TAX-2/-4 channels. Dauer-pheromones and possibly environmental signals (i.e. absence of food, high temperature) inhibit the guanylyl cyclase DAF-11, thus causing dauer-entry by blocking release of ASJ signals. (**b**) ChR2(C128S) was expressed in ASJ sensory neurons using the *trx-1* promoter. *daf-11(m84)*, *daf-11(m84);zxIs19[ptrx-1::TRX-1B::ChR2(C128S)::YFP;lin-15^+^]*, N2;;*zxIs19[ptrx-1::TRX-1B::ChR2(C128S)::YFP;lin-15^+^]*, and N2*; zxEx450[ptrx-1::TRX-1B;pCFJ90]* adults were allowed to lay eggs in the dark for 10–12 hours on plates with bacterial food, supplemented with or without ATR. Plates were then illuminated for 3 days and scored for adult and dauer-animals; n = 10–21 experiments, 30–130 animals each. (**c**) *daf-11(m84)*, *daf-11(m84); zxIs19[ptrx-1::TRX-1B::ChR2(C128S)::YFP;lin-15^+^]*, and *daf-11 (m84); zxEx450[ptrx-1::TRX-1B;pmyo-2::mCherry]* animals were allowed to develop into dauers in the dark on plates optionally supplemented with ATR, and then illuminated beginning at t = 0 h. The fraction of animals escaping dauer-arrest was scored at the indicated times post illumination onset. n = 8–15 experiments, 30–60 animals each. Error bars are s.e.m., ***: p<0.001.

Lastly, we asked whether ChR2(C128S) could sufficiently activate neurons at a timescale of hours to days, e.g. cells relevant for animal development, under low light conditions, to prevent phototoxic effects. We thus turned to neurons that affect larval development. Depending on external signals, *C. elegans* larvae either develop to adulthood, or enter the dauer-state (**Fig. 4a**). In a simplistic view, but based on results from several studies, favorable external signals are sensed by ADF, ASG, and ASI neurons to prevent dauer-entry and to commit the worm to reproductive development [19],[23]. Contrary, ASJ neurons, which can release insulin and possibly other signals to prevent dauer-entry, may rather sense unfavorable cues like pheromones, and thus likely become inhibited [20]. Additionally, ASJ may be involved in dauer-exit, i.e. when conditions become favorable again, by releasing molecules that promote dauer-exit [19]. Unfavorable environmental signals appear to inhibit the guanylyl cyclase DAF-11 (which generates cGMP to activate the cGMP-gated cation-channel TAX-2,-4), thus likely keeping ASJ in a resting or even hyperpolarized state and initiating dauer-arrest [20], [24]–[26] (**Fig. 4a**). *daf-11(m84)* mutants display a constitutive dauer-phenotype (*daf-c*): most larvae become dauers even under favorable conditions [24]. While additional mechanisms affecting dauer larval development need to be considered, (photo-)depolarization of ASJ neurons, at the right time during development, may nonetheless provide a means to prevent dauer-entry and to promote dauer-exit, particularly in *daf-11(m84)* mutants.

To allow cell-specific expression of ChR2(C128S) in ASJ neurons, we used the *trx-1b* promoter, which, however, expresses in ASJ cells only in the context of the genomic locus including the *trx-1b* coding sequences and introns [27]. We thus needed to fuse ChR2(C128S) to the C-terminus of TRX-1B. Expression of TRX-1B::ChR2(C128S) in ASJ was observed after hatching and through all developmental stages, including the dauer stage (**Fig. S5**). To verify that the ChR2 portion of the TRX-1B::ChR2(C128S) fusion protein is functional, we expressed it also in body wall muscle cells, where it could photo-evoke muscle contractions, albeit to a lesser extent than ChR2(C128S) alone (**Fig. S1a, d**). We also (over)expressed only TRX-1B in ASJ cells, to exclude light-independent effects of TRX-1B on dauer-entry or -exit.

Next, we analyzed whether photoactivation of TRX-1B::ChR2(C128S) in ASJ was able to prevent dauer-entry of *daf-11(m84)* mutant animals. To this end, eggs (from mothers that were kept in the dark at all times) were incubated in the presence of bacterial food, optionally supplemented with ATR, under constant low illumination (∼0.12 µW/mm^2^ blue light). After 3 days, we scored the fraction of worms in dauer and adult states. Of the *daf-11(m84)* mutant animals, only 16.5±0.9% or 14.9±1.0% (in the absence or presence of ATR, respectively) developed to adulthood (**Fig. 4b**). However, when TRX-1B::ChR2(C128S)::YFP was expressed in *daf-11(m84)* mutants, photoactivation prevented dauer-formation in 76.4±2.7% of *daf-11(m84)* animals, when ATR was present; a slight positive effect of this transgene was also observed in the absence of ATR (41.4±5.1% adult animals), possibly due to carry-over of some ATR from parental animals. Wild type animals expressing either TRX-1B::ChR2(C128S) or TRX-1B alone yielded close to 100% adult animals, regardless of ATR treatment, and TRX-1B expression in *daf-11(m84)* mutants had no rescuing effect on the dauer-state either (see below, dauer-exit experiment; **Fig. 4c**).

Finally, we investigated whether photoactivation of ASJ could also promote dauer-exit. *daf-11* mutants, optionally expressing TRX-1B::ChR2(C128S) or TRX-1B alone in ASJ, were grown in the dark, in the presence or absence of ATR. Without light-stimulation, animals became dauers, and were only then placed into light, to potentially evoke acute dauer rescue. The fraction of animals developing to adults was scored over 4 days: 2–4% of *daf-11(m84)* dauers and *daf-11(m84)* dauers expressing TRX-1B recovered every 24 h, independent of ATR and light (**Fig. 4c**); yet, within the first 24 h of illumination, 16.4±2.1% of *m84* dauers expressing TRX-1B::ChR2(C128S) in ASJ recovered if ATR was added prior to dauer-entry. At later times, the fraction of adults increased as slowly as for non-transgenic *daf-11(m84)* mutants. Thus, depolarizing ASJ indeed partially induces dauer-exit. However, as this was rather inefficient, additional cells may be required to trigger dauer-exit effectively. Alternatively, as dauers do not feed, ATR may have decayed after the time of dauer entry. We thus analyzed at distinct times after removal from ATR, to what extent ChR2(H134R) and ChR2(C128S) remained functional in muscle cells, by analyzing photo-evoked contractions (**Fig. S6**). While ChR2(H134R) remained fully functional for 24 h, and showed half maximal activity even after 72 h, ChR2(C128S) was surprisingly susceptible to ATR-deprivation: Already after 4 h, functionality was reduced by ∼46%, and essentially non-detectable after 48 h (0.5±0.3% contraction; **Fig. S6**). Importantly, ChR2(C128S) remained fully functional in the presence of ATR even after 72 h (8.7±0.6% contraction; ****). In sum, ChR2(C128S) can be used to alter animal development when expressed in neurons that make developmental decisions, as these can be long-term depolarized using low light intensity.

## Discussion

Using *C. elegans*, we showed that: 1) photoactivation of slow ChR2 variants induces prolonged depolarization in BWMs, cholinergic and GABAergic neurons; 2) ChR2(C128S)-mediated depolarization induced by 1 s light lasts several minutes and requires about one order of magnitude less light than ChR2(H134R); 3) blue-light activated ChR2(C128S) can be inactivated by yellow light, enabling full temporal control, thus neurons can be switched “on” and “off”; 4) photoactivation of ChR2(C128S) in command interneurons evokes long-term behavioral effects; and 5) using ChR2(C128S) even animal development can be altered.

Photoactivating command interneurons evoked long-term behavioral changes. Zheng et al. (1999) reported a largely increased frequency of ∼40 reversals min^−1^ (the “lurcher” phenotype) after expressing constitutively active GLR-1(A687T) AMPARs in command neurons using the same *glr-1* promoter fragment that we used. Thus, upon permanent strong depolarization, neither forward nor backward command neurons gain dominance, in line with mutual inhibition between the two neuron types. However, we observed a reversal right after photoactivation, often persisting for the whole minute, until yellow light closed ChR2(C128S). Regardless of photoactivation, animals exhibited ∼3–4 reversals per minute. How may these opposing results be explained? Unlike ChR2(C128S), GLR-1(A687T) is expressed in its “native environment” (however, in the same cells as ChR2(C128S), as the same promoter was used in both studies), with a likely single-channel conductance in the low pS range, i.e. significantly higher than ChR2 (∼40fS; [28]). Thus, stronger depolarization caused by GLR-1(A/T) might account for different behaviors seen in both experiments. Alternatively, GLR-1(A/T) causes depolarization of command neurons from its earliest expression, thus adaptation may occur, evoking different behaviors, while ChR2(C128S) is acutely induced by light during the experiment. To test this possibility, we photoactivated ChR2(C128S) in command neurons during development and until adulthood, but we found no emerging lurcher phenotype (data not shown). However, as ChR2(C128S) activity dropped to ∼18% during long-term experiments (**Fig. 1f**), we may not achieve a long-lasting depolarization to the same extent as the GLR-1(A/T) channel did [21].

Photoactivation of TRX-1B::ChR2(C128S)::YFP in ASJ sufficed to depolarize these neurons for hours, allowing effective dauer-rescue of *daf-11(m84)* mutants. We verified that these effects are specific for the ChR2(C128S) portion of the fusion protein, and that TRX-1B alone had no effects; furthermore, as reported previously, mutation of *trx-1* caused neither *daf-c* nor *daf-d* phenotypes [27]. The photo-evoked dauer rescue was 76.4±2.7%, however, it was not 100%: This may indicate that ChR2(C128S)-induced depolarization was insufficient in some animals, or that additional cellular mechanisms affect dauer-entry in *daf-11* mutants, which could not be overcome by ASJ photodepolarization; clearly the dauer developmental pathway involves many more cells expressing DAF-11 (e.g. ASI, ASK, AWB, and AWC) than just ASJ, and complex signaling pathways that may only inefficiently be triggered via simple depolarization of ASJ neurons. ASJ also promoted dauer-exit in a small, but significant fraction of animals during a 24 h photoactivation period (16.4±2.1%). Possibly, additional neurons, not photoactivated, need to cooperate with ASJ to promote dauer-exit more effectively; yet, more likely, ASJ was insufficiently depolarized due to progressive ChR2(C128S) inactivation, and due to the observed susceptibility of ChR2(C128S) to ATR-deprivation. Nevertheless, to prevent dauer-entry, ASJ is highly efficient on its own.

ChR2(C128S) has some critical properties that should be considered when designing experiments. One is the dependence on the continuous presence of ATR, the other is the partial inactivation by repeated or premature activation. For example, this makes ChR2(C128S) ill-suited for electrophysiological measurements in *C. elegans*, which require dissection of the animals under intense white light, which appears to render a majority of ChR2(C128S) to decay to “lost” states (hence the small photocurrents measured in **Fig. 1c**).

Nonetheless, ChR2(C128S) complements present optogenetic tools and expands their field of application, conceivably also in other animal models. Additional developmental pathways may now be probed, e.g. the likely activity-dependent polarity changes of *C. elegans* DD motorneurons during development [29]. Also adaptation or even associative learning within sensory circuits, which involves long-term neuronal activation [30], may be subjected to optogenetic manipulation using ChR2(C128S).

## Materials and Methods

### *C. elegans* culture and transgenic animals

Wild-type and mutant strains used originate from the Bristol strain N2 [31]. Strains were cultivated, optionally in the presence of all-*trans* retinal (Sigma-Aldrich), as described previously [5]. Microinjection of DNA was performed according to standard protocols using 80–100 ng/µl (for constructs with *Pmyo-3* and *Punc-17*), or 200 ng/µl (*Pglr-1* and *Ptrx-1*), and 80 ng/µl for the marker lin-15^+^ or 2.5 ng/µl for the marker pCFJ90 (*pmyo-2::mCherry*), respectively. The *zxEx434[ptrx-1::TRX-1B::ChR2(C128S)::YFP;lin-15^+^]* transgene was genomically integrated via UV-irradiation.

Strains used were: **ZX299:** *lin-15(n765ts^−^);zxEx22[pmyo-3::ChR2(H134R)::YFP;lin-15^+^]* [4], **ZX426:** *N2;zxIs3[punc-47::ChR2(H134R)::YFP;lin-15^+^*] [5], **ZX460:** N2;*zxIs6[punc-17::ChR2(H134R)::YFP;lin-15^+^]* [5], **ZX497:** *unc-47(e407);zxIs6[punc-17::ChR2(H134R)::YFP;lin-15^+^]* [5], **ZX836:** *lin-15(n765ts^-^);zxEx421[pmyo-3::ChR2(C128A)::YFP;lin-15^+^]*, **ZX837:** *lin-15(n765ts^-^);zxEx422[pmyo-3::ChR2(C128T)::YFP;lin-15^+^]*, **ZX838:** *lin-15(n765ts^-^);zxEx423[pmyo-3::ChR2(C128S)::YFP;lin-15^+^]*, **ZX839:** *lin-15(n765ts^-^);zxEx424[punc-17::ChR2(C128A)::YFP;lin-15^+^]*, **ZX840:** *lin-15(n765ts^-^);zxEx425[punc-17::ChR2(C128T)::YFP;lin-15^+^]*, **ZX841:** *lin-15(n765ts^-^);zxEx426[punc-17::ChR2(C128S)::YFP;lin-15^+^]*, **ZX842:** *unc-49(e407);lin-15(n765ts^-^);zxEx424[punc-17::ChR2(C128A)::YFP;lin-15^+^]*, **ZX843:** *unc-49(e407);lin-15(n765ts^-^);zxEx425[punc-17::ChR2(C128T)::YFP;lin-15^+^]*, **ZX844:** *unc-49(e407);lin-15(n765ts^-^);zxEx426[punc-17::ChR2(C128S)::YFP;lin-15^+^]*, **ZX845:** *lin-15(n765ts^-^);zxEx428[punc-47::ChR2(C128A)::YFP;lin-15^+^]*, **ZX846:** *lin-15(n765ts^-^);zxEx429[punc-47::ChR2(C128T)::YFP;lin-15^+^]*, **ZX847:** *lin-15(n765ts^-^),zxEx430[punc-47::ChR2(C128S)::YFP;lin-15^+^]*, **ZX848:** *lin-15(n765ts^-^);zxEx431[pglr-1::ChR2(H134R)::YFP;lin-15^+^]*, **ZX849:** *lin-15(n765ts^-^);zxEx432[pglr-1::ChR2(C128S)::YFP;lin-15^+^]*, **ZX851:** *lin-15(n765ts^-^);zxEx434[ptrx-1::TRX-1B::ChR2(C128S)::YFP;lin-15^+^]*, **ZX852:** *N2;zxIs19[ptrx-1::TRX-1B::ChR2(C128S)::YFP;lin-15^+^]*, **ZX884:** *daf-11(m84);zxIs19[ptrx-1::TRX-1B::ChR2(C128S)::YFP;lin-15^+^]*, **ZX1033:** *lin-15(n765ts^-^);zxEx448[pmyo-3::TRX-1B::ChR2(C128S)::YFP;lin-15^+^]*, **ZX1034:** *N2;zxEx450[ptrx-1::TRX-1B;pmyo-2::mCherry]*, **ZX1035:** *daf-11(m84); zxEx450[ptrx-1::TRX-1B;pmyo-2::mCherry]*

### Molecular biology

GenBank accession of ChR2 is AF461397. Plasmids pCS54(*Pmyo-3::ChR2(C128A)::YFP*) and pCS56(*Pmyo-3::ChR2(C128T)::YFP*) were obtained by exchanging fragments including mutations from pGEMHE-ChR2(C128A) and pGEMHE-ChR2(C128T) into pAG54(*Pmyo-3::ChR2(H134R)aa1-310::YFP*) [4] via StuI/XhoI. pCS86(*pmyo-3::ChR2(C128S)::YFP*) was generated from pCS54 by site-directed mutagenesis. For expression in cholinergic neurons using *Punc-17*, BglII/StuI fragments were transferred from plasmids pCS54, pCS56 and pCS86 to *Punc-17::ChR2(H134R)::YFP* [5] resulting in pCS55(*Punc-17::ChR2(C128A)::YFP*), pCS57(*Punc-17::ChR2(C128T)::YFP*) and pCS87(Punc-17::ChR2(C128S)::YFP). Likewise, StuI/XhoI fragments were swapped from pCS54, pCS56 and pCS86 into *Punc-47::ChR2(H134R)::YFP* [5], yielding pCS124(*Punc-47::ChR2(C128A)::YFP*), pCS125(*Punc-47::ChR2(C128T)::YFP*) and pCS126(*Punc-47::ChR2(C128S)::YFP*). For expression in command interneurons, a *Pglr-1* fragment was PCR-amplified from genomic DNA (primers oCS209(5′-GTGTCACGTGCCATGATTACGCCAAGCTTGC-3′) and oCS210(5′-CAATCCCGGGGATCCTCTAG-3′)), and subcloned into pAG54 and pCS86 using PmlI/BamHI, yielding pCS103(*Pglr-1::ChR2(H134R)::YFP*) and pCS106(*Pglr-1::ChR2(C128S)::YFP*). The *Ptrx-1::TRX-1B* sequence for expression in ASJ was PCR-amplified from genomic DNA (oCS211(5′-GTGTCACGTGAGAATGGATACCTGATCATT-3′) and oCS224(5′-GTGTGGATCCTTGAGCAGATACGTGCTCC-3′)). A PmlI/BamHI fragment was exchanged in pCS86 yielding pCS121(*Ptrx-1::TRX-1B::ChR2(C128S)::YFP*). A fragment was amplified from pCS121 using primers oCS269(5′-GTGTTCTAGAATGTCTCTCACCAAGGAG-3′) and oCS270(5′-GAGAATGACCGGTGAGG-3') and subcloned into pCS86 with XbaI and XhoI to result in pCS155(*Pmyo-3::TRX-1B::ChR2(C128S)::YFP*). In pCS121, *ChR2(C128S)::YFP* was excised with BglII and EcoRI and replaced by a PCR fragment from pCS121 (primers oCS271(5′-GGTAATTCGGTAAAACTC-3′) and oCS272(5′-CACAGAATTCTCATTGAGCAGATACGTGCTCC-3′) to generate pCS156(*Ptrx-1::TRX-1B*).

### Behavioral experiments

Young adult animals were transferred to 5.5 cm dishes containing 4 ml nematode growth medium (NGM). Using an Axiovert 40 CFL microscope (Zeiss) with 10× magnification, 50 W mercury lamp, and computer-controlled shutter (Sutter Instruments), animals were illuminated with 450–490 nm blue light for ChR2 photoactivation and with 565–595 nm yellow light for ChR2 photoinactivation. Intensity was adjusted using neutral density filters (AHF Analysentechnik). For long-term photoactivation and inactivation, LEDs, blue (470 nm; 0.05 mW/mm^2^; Luxeon) or yellow (590 nm; 0.04 mW/mm^2^; Rapp Optoelectronic), respectively, were used. For body length measurements, videos were recorded (Powershot G5 or G9 digital cameras, Canon). Frames were extracted and either processed using a custom ImageJ script [32] or analyzed with a custom script for Matlab (The MathWorks) [5]. Unless described differently, animals were kept in complete darkness until execution of experiments to avoid unwanted photoactivation of ChR2. To avoid coiling induced by prolonged depolarization of cholinergic neurons [5], we analyzed effects of ChR2 activation in *unc-49(e407)* mutants, lacking GABA_A_Rs. Body length was normalized to the last second before illumination. Images yielding incorrect values for body length (e.g. coiling animals) were ignored. To monitor effects on dauer-entry, the following strains were cultivated for at least three days in the dark: *daf-11(m84)*, N2, ZX852, ZX884, and ZX1034. Then, young adults were placed on seeded plates, optionally supplemented with ATR while plates were exposed to continuous illumination of two 18 W neon bulbs for three days (blue light intensity: 0.12 µW/mm^2^ at the NGM agar surface). Animals were allowed to lay eggs for 10–12 h, and then removed. The fraction of adults and dauers (grown with or without ATR) was scored. To analyze dauer-exit, the following strains were cultivated on seeded plates with or without ATR for at least two days in the dark (to enlarge the fraction of dauer-animals): *daf-11(m84)*, ZX884, and ZX1035. Dauer animals were then transferred to fresh plates, optionally supplemented with ATR and incubated under constant illumination (two 18 W neon bulbs; 0.12 µW/mm^2^ blue light intensity). The fraction of adults was then scored daily.

### Fluorescence microscopy and Electrophysiology

Expression of ChR2::YFP was analyzed on an Axiovert 200 microscope (Zeiss) with filterset F41-028 (AHF Analysentechnik) and 100 W mercury lamp. Images were captured with an AxioCam MRm camera (Zeiss). Expression in command interneurons and ASJ was analyzed on a Zeiss LSM 510 confocal microscope. Recordings from BWMs were conducted as previously described [5].

### Statistics

Data are given as means±s.e.m. Significance between datasets is given as P-value after two-tailed Student's t-test.

## Supporting Information

Figure S1 **Expression and activation of slow ChR2 variants in body wall muscle cells evokes body contractions.** (**a**) ChR2(H134R)::YFP, ChR2(C128T)::YFP, ChR2(C128A)::YFP, ChR2(C128S)::YFP, and TRX-1B::ChR2(C128S)::YFP were expressed in body wall muscle cells using the *myo-3* promoter. Fluorescence micrographs. Scale bar is 10 µm. (**b**) Relative body length of animals expressing ChR2(C128S) while a low-intensity 1 s blue light stimulus (0.01 mW/mm^2^; 450–490 nm) was given at t = 0 s. (**c**) Enlarged diagram from (b) ranging from −1–5 s, comparing full contractions evoked by ChR2(C128S) to largely reduced contractions evoked by ChR2(H134R). (**d**) Relative body length of worms expressing ChR2(C128S) or TRX-1B::ChR2(C128S) while 1s blue (1.4 mW/mm^2^; 450–490 nm) or 1 s yellow (4.4 mW/mm^2^; 565–595 nm) light pulses were presented, as indicated. Shown are means, error bars are s.e.m.; n = number of animals.
(TIF)

Figure S2 **Photoactivation and -inactivation of ChR2(C128S) in cholinergic motorneurons.** Repeated activation and inhibition of ChR2(C128S) in cholinergic neurons, using blue and yellow light pulses, as indicated. The body contractions are shown as readout for postsynaptic muscle activation, induced by photo-triggered release of acetylcholine from motorneurons. Shown are mean relative body length and s.e.m.; n = number of animals.
(TIF)

Figure S3 **Prolonged depolarization of GABAergic motorneurons via ChR2(C128S).** ChR2(H134R) and ChR2(C128S) were expressed in GABAergic motorneurons using the *unc-47* promoter. Body length and the consequent elongation were measured as readout for presynaptic GABA release. (**a**) Mean relative body length of animals while a 1 s blue light stimulus (2.1 mW/mm^2^; 450–490 nm) was given at t = 0. (**b**) mean relative body length of animals while alternating 1 s blue (2.1 mW/mm^2^; 450–490 nm) or 1 s yellow (6.1 mW/mm^2^; 565–595 nm) light pulses were presented. n = number of animals; error bars are s.e.m.; blue and yellow bars indicate the duration of illumination with the respective color of light.
(TIF)

Figure S4 **ChR2(C128S) expression in command interneurons and other neurons, using the** ***Pglr-1*** **promoter.** ChR2(C128S)::YFP was expressed in command interneurons (AVA, AVB, AVD, AVE, PVC) and other cells (AIB, RMD, RIM, SMD, AVG, PVQ, URY) using the *glr-1*-promoter (Maricq et al., 1995, Nature 378:78–81). Confocal z-projection (left) and bright-field image (right). Scale bar = 30 µm.
(TIF)

Figure S5 **TRX-1B::ChR2(C128S) expression in ASJ sensory neurons.** Shown is a *daf-11(m84)* dauer larva expressing TRX-1B::ChR2(C128S)::YFP in ASJ sensory neurons using the *trx-1* promoter. Dendrites are indicated by arrows, arrowheads point to axons in the nerve ring. Confocal z-projection (left) and bright-field image (right). Scale bar = 30 µm.
(TIF)

Figure S6 **Long-term activity test of ChR2(C128S) and ChR2(H134R) in muscle cells of animals removed from ATR plates.** Animals expressing ChR2(H134R) or ChR2(C128S) in muscle cells were cultivated on ATR. At larval stage L4, worms were transferred to fresh plates either with or without ATR. At regular intervals blue light (1.4 mW/mm^2^; 450–490 nm) was presented and resulting contractions were measured. Shown are means, error bars are s.e.m.; n = number of animals.
(TIF)

Video S1 **Photoactivation and –inactivation of ChR2(C128S) in body wall muscle cells.** Photoactivation of ChR2(C128S) in body wall muscle cells with blue light (1 s; 450–490 nm; 0.01 mW/mm^2^) caused contraction and was terminated with yellow light (1 s; 565–595 nm; 4.4 mW/mm^2^); 15 frames per second.
(MOV)

Video S2 **Photoactivation and –inactivation of ChR2(C128S) in cholinergic motorneurons.** Photoactivation of ChR2(C128S) in cholinergic motorneurons with blue light (1 s; 450–490 nm; 0.01 mW/mm^2^) caused contraction and was terminated with yellow light (1 s; 565–595 nm; 2.5 mW/mm^2^); 15 frames per second.
(MOV)

Video S3 **Photoactivation and –inactivation of ChR2(C128S) in command interneurons.** Photoactivation of ChR2(C128S) in command interneurons with blue light (450–490 nm; 2.1 mW/mm^2^) induced backward movement and is reversed by yellow light (565–595 nm; 6.1 mW/mm^2^); 15 frames per second.
(MOV)
