# Supplementary figures and images for: Optogenetic Long-Term Manipulation of Behavior and Animal Development
Source: PLoS One. 2011 Apr 20;6(4):e18766. doi: 10.1371/journal.pone.0018766 (PMC3080377; doi:10.1371/journal.pone.0018766)

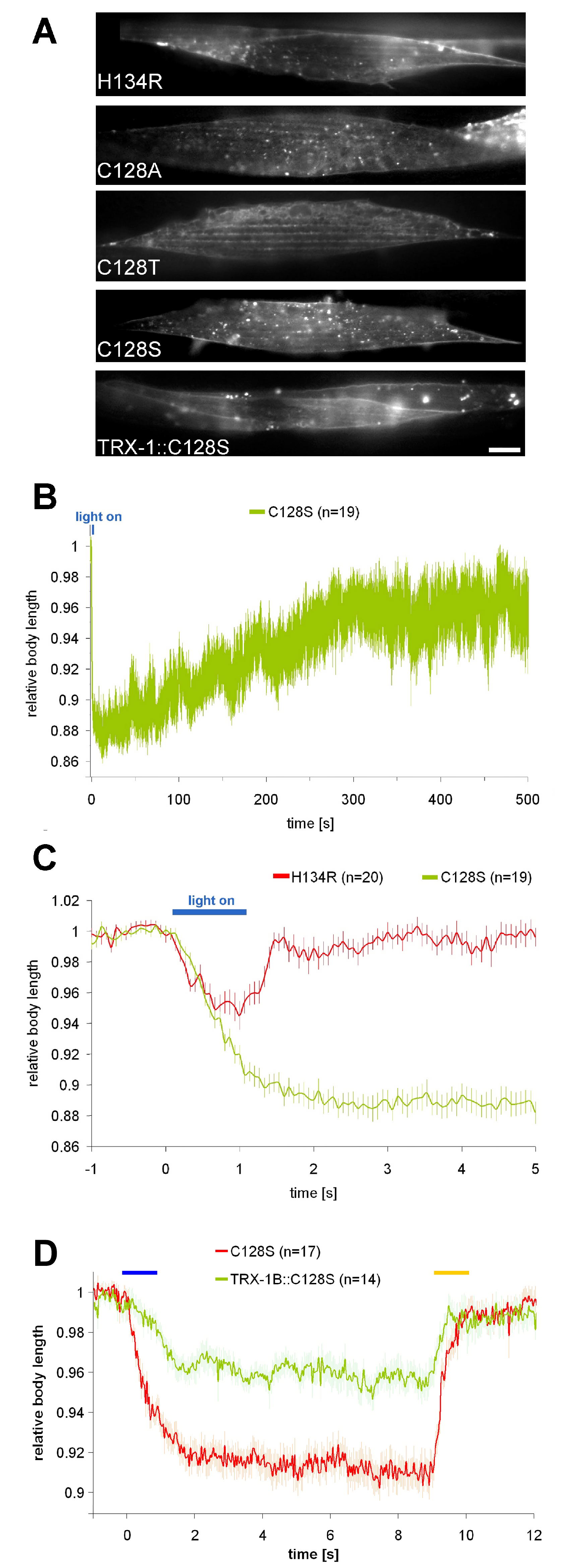

Supplement: Figure S1 — Expression and activation of slow ChR2 variants in body wall muscle cells evokes body contractions. (a) ChR2(H134R)::YFP, ChR2(C128T)::YFP, ChR2(C128A)::YFP, ChR2(C128S)::YFP, and TRX-1B::ChR2(C128S)::YFP were expressed in body wall muscle cells using the myo-3 promoter. Fluorescence micrographs. Scale bar is 10 µm. (b) Relative body length of animals expressing ChR2(C128S) while a low-intensity 1 s blue light stimulus (0.01 mW/mm2; 450–490 nm) was given at t = 0 s. (c) Enlarged diagram from (b) ranging from −1–5 s, comparing full contractions evoked by ChR2(C128S) to largely reduced contractions evoked by ChR2(H134R). (d) Relative body length of worms expressing ChR2(C128S) or TRX-1B::ChR2(C128S) while 1s blue (1.4 mW/mm2; 450–490 nm) or 1 s yellow (4.4 mW/mm2; 565–595 nm) light pulses were presented, as indicated. Shown are means, error bars are s.e.m.; n = number of animals. (TIF) [file pone.0018766.s001.tif]

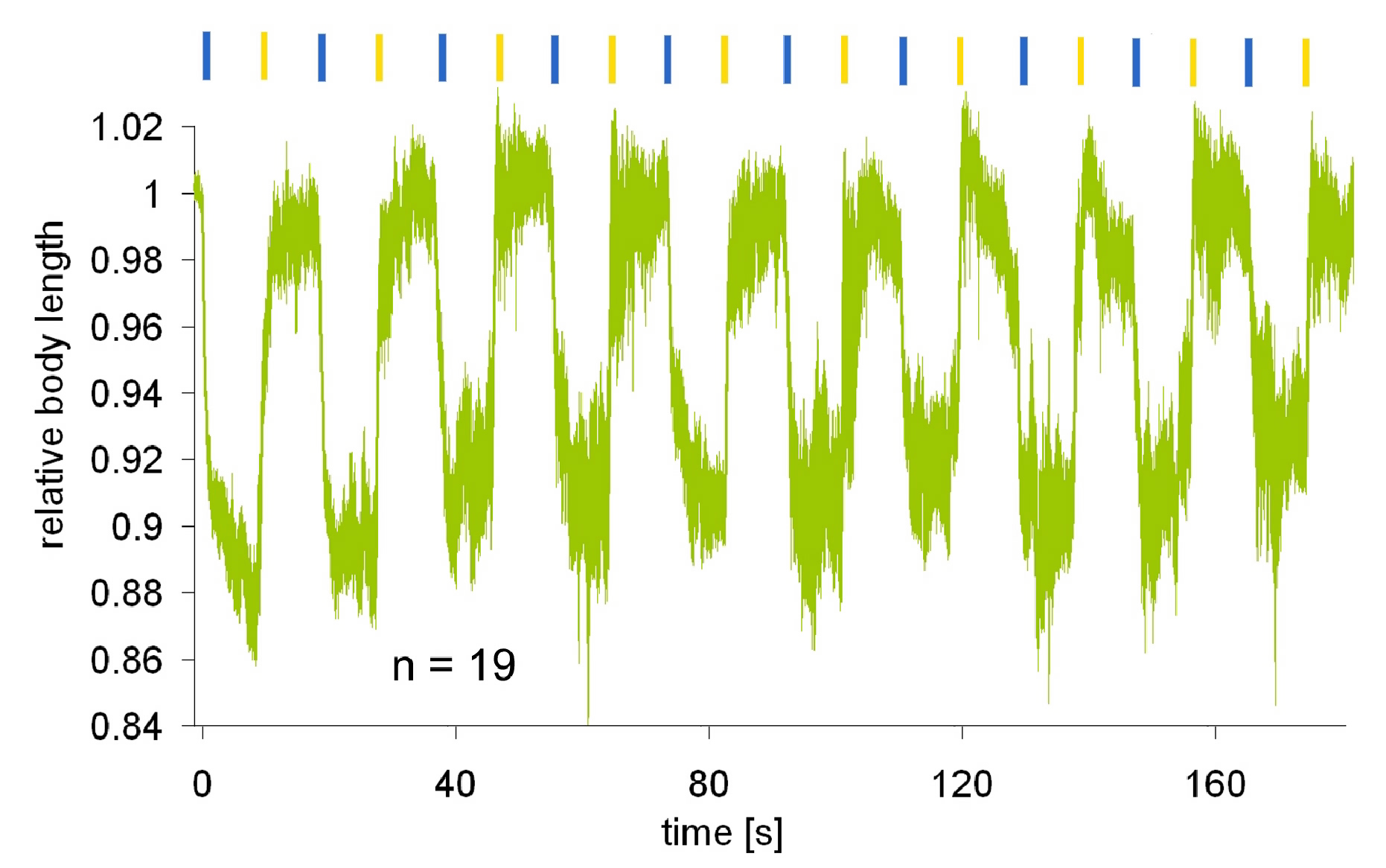

Supplement: Figure S2 — Photoactivation and -inactivation of ChR2(C128S) in cholinergic motorneurons. Repeated activation and inhibition of ChR2(C128S) in cholinergic neurons, using blue and yellow light pulses, as indicated. The body contractions are shown as readout for postsynaptic muscle activation, induced by photo-triggered release of acetylcholine from motorneurons. Shown are mean relative body length and s.e.m.; n = number of animals. (TIF) [file pone.0018766.s002.tif]

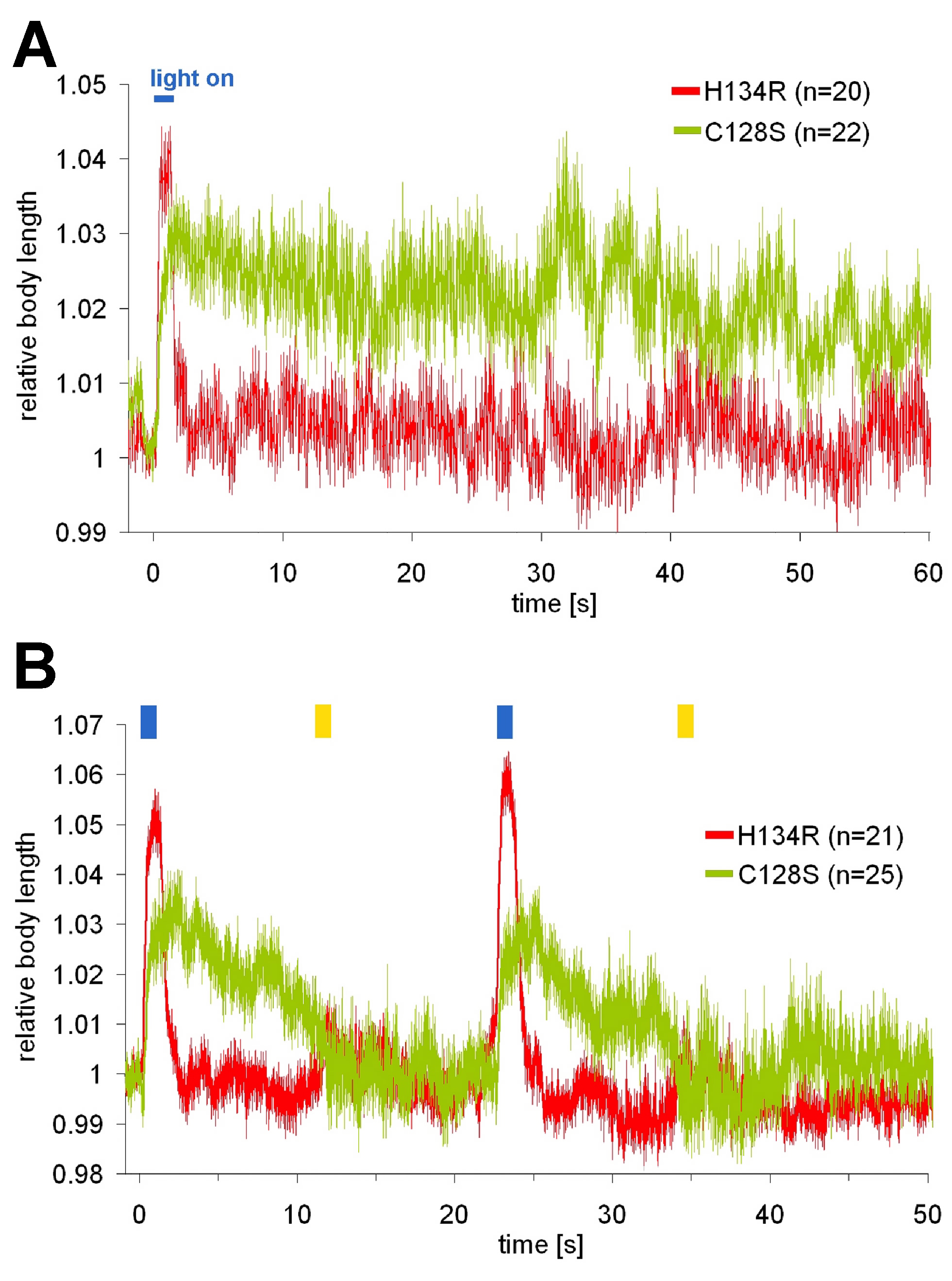

Supplement: Figure S3 — Prolonged depolarization of GABAergic motorneurons via ChR2(C128S). ChR2(H134R) and ChR2(C128S) were expressed in GABAergic motorneurons using the unc-47 promoter. Body length and the consequent elongation were measured as readout for presynaptic GABA release. (a) Mean relative body length of animals while a 1 s blue light stimulus (2.1 mW/mm2; 450–490 nm) was given at t = 0. (b) mean relative body length of animals while alternating 1 s blue (2.1 mW/mm2; 450–490 nm) or 1 s yellow (6.1 mW/mm2; 565–595 nm) light pulses were presented. n = number of animals; error bars are s.e.m.; blue and yellow bars indicate the duration of illumination with the respective color of light. (TIF) [file pone.0018766.s003.tif]

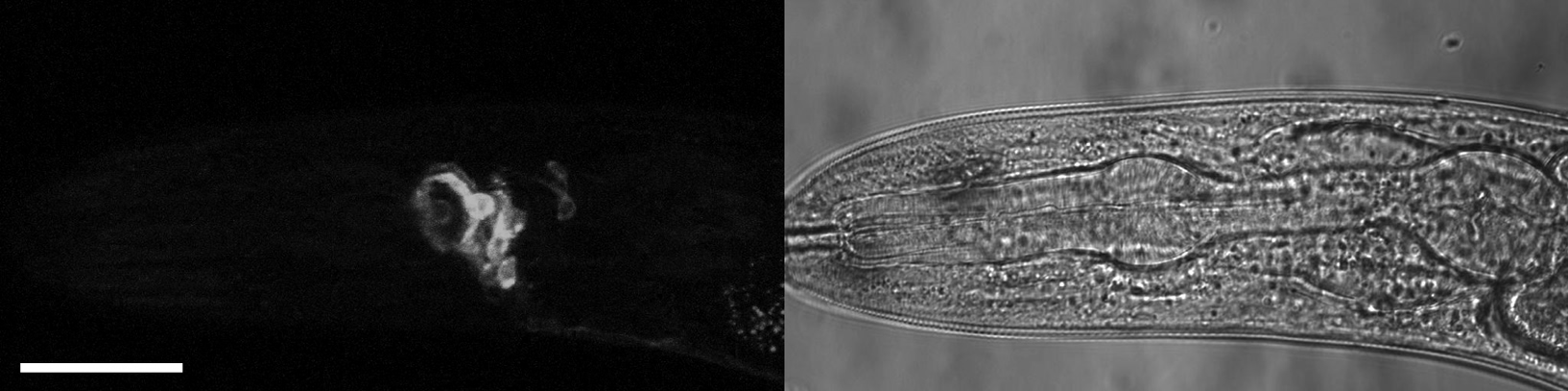

Supplement: Figure S4 — ChR2(C128S) expression in command interneurons and other neurons, using the Pglr-1 promoter. ChR2(C128S)::YFP was expressed in command interneurons (AVA, AVB, AVD, AVE, PVC) and other cells (AIB, RMD, RIM, SMD, AVG, PVQ, URY) using the glr-1-promoter (Maricq et al., 1995, Nature 378:78–81). Confocal z-projection (left) and bright-field image (right). Scale bar = 30 µm. (TIF) [file pone.0018766.s004.tif]

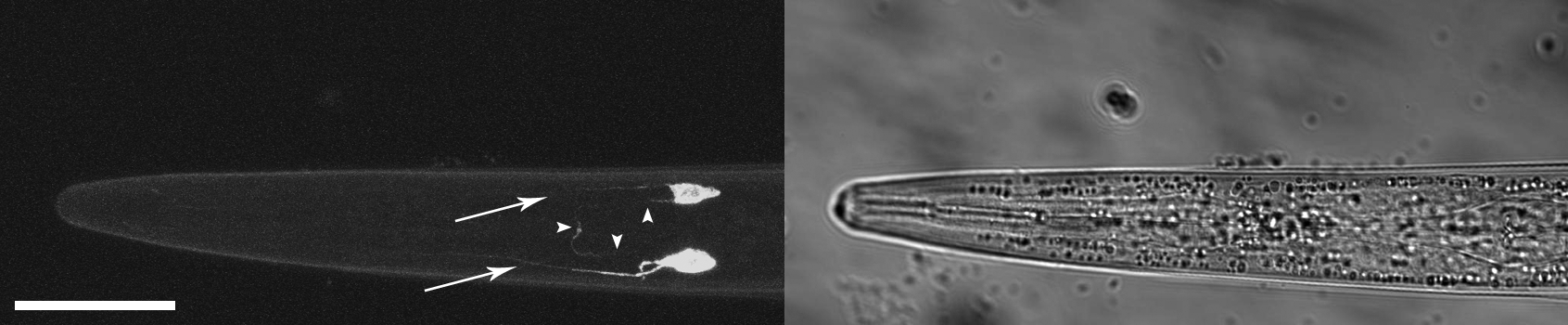

Supplement: Figure S5 — TRX-1B::ChR2(C128S) expression in ASJ sensory neurons. Shown is a daf-11(m84) dauer larva expressing TRX-1B::ChR2(C128S)::YFP in ASJ sensory neurons using the trx-1 promoter. Dendrites are indicated by arrows, arrowheads point to axons in the nerve ring. Confocal z-projection (left) and bright-field image (right). Scale bar = 30 µm. (TIF) [file pone.0018766.s005.tif]

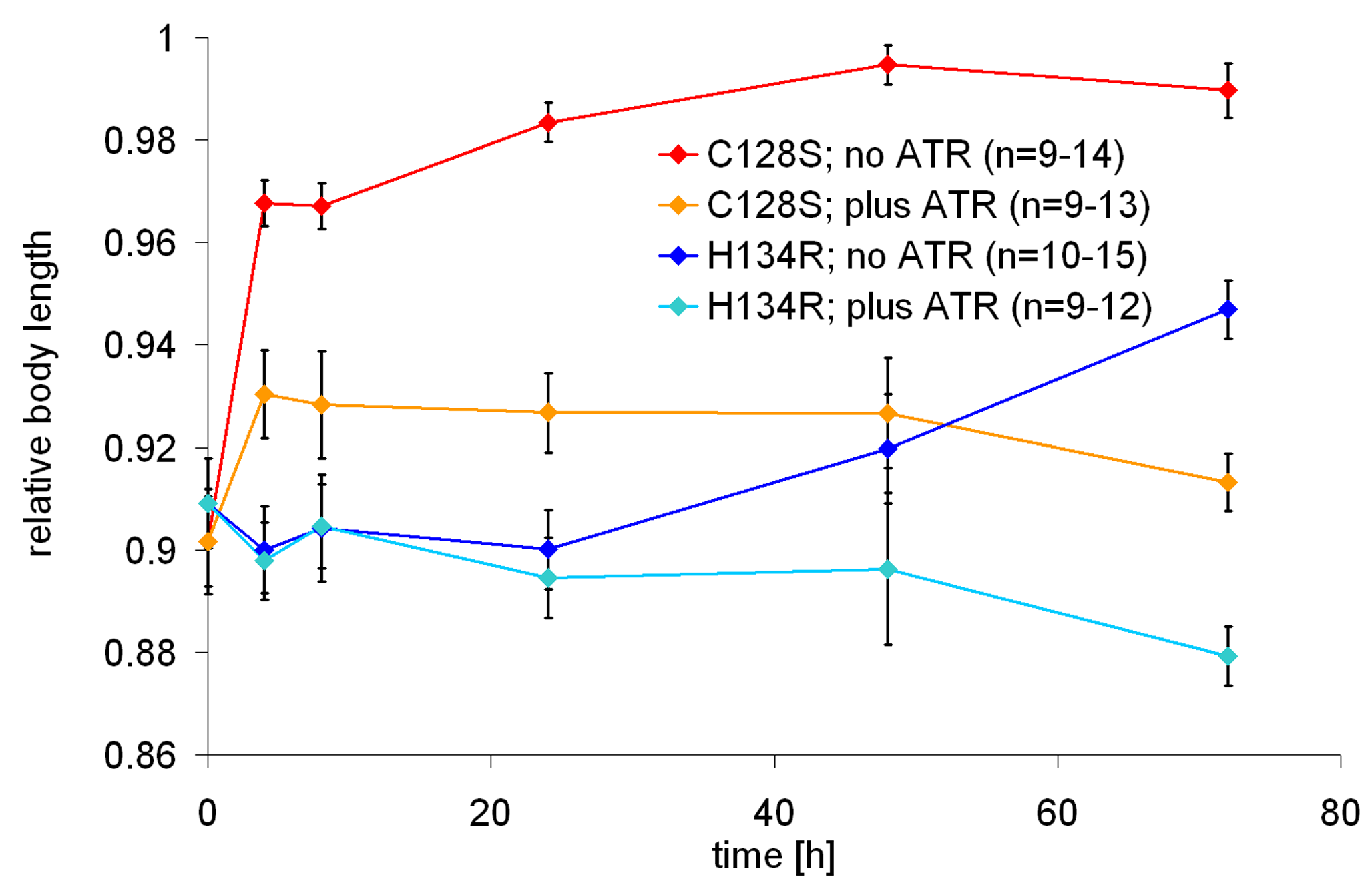

Supplement: Figure S6 — Long-term activity test of ChR2(C128S) and ChR2(H134R) in muscle cells of animals removed from ATR plates. Animals expressing ChR2(H134R) or ChR2(C128S) in muscle cells were cultivated on ATR. At larval stage L4, worms were transferred to fresh plates either with or without ATR. At regular intervals blue light (1.4 mW/mm2; 450–490 nm) was presented and resulting contractions were measured. Shown are means, error bars are s.e.m.; n = number of animals. (TIF) [file pone.0018766.s006.tif]
